# *Amblyomma cajennense* (Fabricius, 1787) (Acari: Ixodidae), the Cayenne tick: phylogeography and evidence for allopatric speciation

**DOI:** 10.1186/1471-2148-13-267

**Published:** 2013-12-09

**Authors:** Lorenza Beati, Santiago Nava, Erica J Burkman, Darci M Barros-Battesti, Marcelo B Labruna, Alberto A Guglielmone, Abraham G Cáceres, Carmen M Guzmán-Cornejo, Renato León, Lance A Durden, João LH Faccini

**Affiliations:** 1 Institute for Coastal Plain Sciences and Biology Department, Georgia Southern University, P.O. Box 8056, Statesboro, GA 30460, USA; 2 Instituto Nacional de Tecnología Agropecuaria, Estación Experimental Agropecuaria Rafaela, CC 22, CP 2300 Rafaela, Santa Fe, Argentina; 3 Department of Infectious Diseases, University of Georgia College of Veterinary Medicine, 501 D.W. Brooks Drive Athens, GA 30602, USA; 4 Laboratório de Parasitologia, Instituto Butantan, Av. Vital Brasil 1500, 05503-900 São Paulo, SP, Brazil; 5 Departamento de Medicina Veterinária Preventiva e Saúde Animal, Faculdade de Medicina Veterinária e Zootecnia, Universidade de São Paulo, São Paulo, SP, 05508-270, Brazil; 6 Departamento Académico de Microbiologia Médica, Facultad de Medicina, Universidad Nacional Mayor de San Marcos, Lima, Perú; 7 Laboratorio de Entomología, Instituto Nacional de Salud, Lima, Perú; 8 Laboratorio de Acarología, Departamento de Biología Comparada, Facultad de Ciencias, Universidad Nacional Autónoma de México, Coyoacán 04510, Distrito Federal, México; 9 Laboratorio de Entomología Médica y Medicina Tropical (LEMMT), Colegio de Ciencias Biológicas y Ambientales, Universidad San Francisco de Quito, Cumbayá, Quito, Ecuador; 10 Biology Department, Georgia Southern University, P.O. Box 8042, Statesboro, GA 30460, USA; 11 Departamento de Parasitologia Animal, Instituto de Veterinária, Universidade Federal Rural do Rio de Janeiro, 23890-000 Seropédica, RJ, Brazil

## Abstract

**Background:**

*Amblyomma cajennense* F. is one of the best known and studied ticks in the New World because of its very wide distribution, its economical importance as pest of domestic ungulates, and its association with a variety of animal and human pathogens. Recent observations, however, have challenged the taxonomic status of this tick and indicated that intraspecific cryptic speciation might be occurring. In the present study, we investigate the evolutionary and demographic history of this tick and examine its genetic structure based on the analyses of three mitochondrial (12SrDNA, d-loop, and COII) and one nuclear (ITS2) genes. Because *A. cajennense* is characterized by a typical trans-Amazonian distribution, lineage divergence dating is also performed to establish whether genetic diversity can be linked to dated vicariant events which shaped the topology of the Neotropics.

**Results:**

Total evidence analyses of the concatenated mtDNA and nuclear + mtDNA datasets resulted in well-resolved and fully congruent reconstructions of the relationships within *A. cajennense*. The phylogenetic analyses consistently found *A. cajennense* to be monophyletic and to be separated into six genetic units defined by mutually exclusive haplotype compositions and habitat associations. Also, genetic divergence values showed that these lineages are as distinct from each other as recognized separate species of the same genus. The six clades are deeply split and node dating indicates that they started diverging in the middle-late Miocene.

**Conclusions:**

Behavioral differences and the results of laboratory cross-breeding experiments had already indicated that *A. cajennense* might be a complex of distinct taxonomic units. The combined and congruent mitochondrial and nuclear genetic evidence from this study reveals that *A. cajennense* is an assembly of six distinct species which have evolved separately from each other since at least 13.2 million years ago (Mya) in the earliest and 3.3 Mya in the latest lineages. The temporal and spatial diversification modes of the six lineages overlap the phylogeographical history of other organisms with similar extant trans-Amazonian distributions and are consistent with the present prevailing hypothesis that Neotropical diversity often finds its origins in the Miocene, after the Andean uplift changed the topology and consequently the climate and ecology of the Neotropics.

## Background

**Figure 1:**
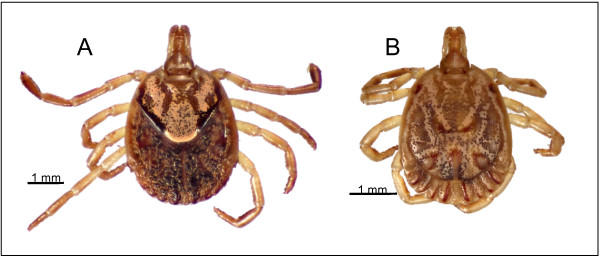
**The Cayenne tick.(A)** Female and **(B)** male *A. cajennense* (dorsal view) from the type locality in French Guiana (RML 124079).

**Figure 2:**
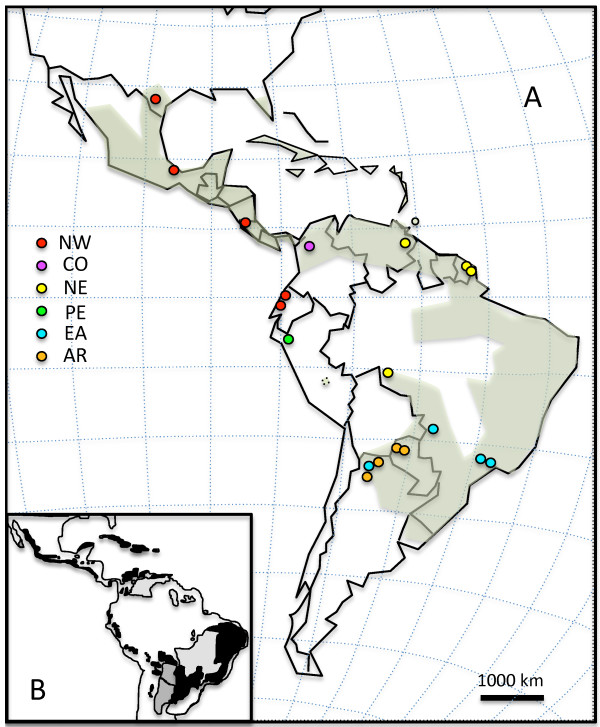
**Known geographical distribution of*A. cajennense* and collection sites.(A)** Shaded green areas represent the approximate known range of *A. cajennense* (modified from [1,40]). Colored circles indicate collection sites. Different colors correspond to the subdivision of the samples into phylogenetic clades as defined by the legend within the figure: NW = north-western clade (Texas, Mexico, Costa Rica, Ecuador), CO = Colombia; NE = north-eastern clade (French Guiana, Brazil-Rondonia); PE = Perú (Inter-Andean valley); EA = eastern clade (Brazil - Atlantic Forest - Mato Grosso and Argentina - Yungas); AR = Argentine clade (Argentine and Paraguayan Chaco). **(B)** Geographical area representing SDTF refugia (in black), savannas (in clear grey) and Chaco in dark grey (modified from [29]).

*Amblyomma cajennense* Fabricius (Figure 1) is one of the most widely distributed tick species in the New World. Its range extends from northern Argentina, to the Caribbean and the southernmost part of the U.S. (from 27° N to 29° S). Throughout its distribution, this tick has adapted to widely different ecological conditions, including ecosystems as different as semi-arid grasslands and subtropical secondary forests [1]. The geographical area occupied by this tick is interspersed with major geographical barriers: the Andes, the Gulf of Mexico, and large rivers [1,2] (Figure 2A).

Early morphological studies resulted in contradicting opinions about the taxonomy of *A. cajennense*. Some authors considered phenotypic differences (presence and number of ventral plates, proportional length of festoons, ornamentation, scutal size and shape, punctation, and shape of porose areas) to identify different species within *A. cajennense*[3-5], whereas other authors ascribed morphological differences to mere intraspecific polymorphism [6-9]. The latter point of view prevailed and, since 1953 [8], all related species were relegated to synonyms of *A. cajennense*. Lack of variation in the cuticular hydrocarbons reported from twelve geographically distinct populations of *A. cajennense* supported the synonymy [10].

Nevertheless, after observing different distinct developmental features (lengths of feeding, pre-molting, pre-ovoposition, and egg incubation periods) in laboratory colonies of *A. cajennense* from different Neotropical areas (Argentina, Brazil, Cuba, U.S., and Trinidad), Guglielmone et al. [11] suggested that *A. cajennense* might be a complex of sibling species. More recently, biological crossbreeding incompatibility was observed between colonies of *A. cajennense* from different geographical areas [12,13]. Unsuccessful crossmating experiments constitute a strong indication of the occurrence of different species. Nevertheless, laboratory breeding conditions may not fully represent natural settings and may artificially impede mating processes that would normally occur in nature.

In order to further investigate these apparently contradicting findings it was therefore necessary to use molecular methods as additional tools for developing better informed taxonomic opinions. Molecular sequence analyses were also used to evaluate the phylogeographical evolutionary history of the taxon as its present distribution, similar to that of other Neotropical organisms [14-22], can contribute to better understand the respective influence of Pleistocene versus Miocene biogeographical events in generating diversity in the Neotropics.

The present distribution of taxa and their diversity is the result of past demographic events such as colonization, expansion, and extinction, usually driven by habitat modifications. The evolution and population differentiation in ticks, obligate blood-sucking ectoparasites of vertebrates, are shaped by two main driving forces: biogeographical vicariance and host association [23-26].

In terms of vicariance, in the Neotropical area, the richness in species diversity had initially been attributed to habitat fragmentation due to the fluctuations in levels of dryness [27] or temperature [28] during the Pleistocene. By degrees, the scientific community realized that the diversification of organisms in the Neotropics could not be solely be attributed to the effect of Quaternary glaciation on Neotropical climate [29-34], but was likely to have started earlier under the effect of the Andean orogenesis [35]. More recently, particular attention has been given to vicariant trans-Amazonian taxa distribution. The geographical range of such organisms often overlaps the remaining nuclei of the Neotropical Seasonally Dry Tropical Forest (SDTF) and of the adjoining dry savannas of the Chaco and Cerrado regions, which are thought to have been widespread during the Neogene [15,29-31,36-38]. The distribution of the Cayenne tick [1] roughly coincides with a typical trans-Amazonian distribution and significantly overlaps SDTF, Chaco and Cerrado refugial foci (Figure 2B) [29,39]. Ticks collected in deeper Amazonian areas (i. e. Rondonia) usually inhabit drier corridors and are found at the interface between forest and savanna and not in the thick of the rain forest [40]. One can, therefore, hypothesize, that like many other organisms with a trans-Amazonian distribution, the Cayenne tick has undergone allopatric speciation in the different ecological regions which resulted from the fragmentation of SDTF and of environments drier and more markedly seasonal than rain forest habitats. However, if we consider host association as the main driving mechanism underlying tick diversification, there is no doubt that *A. cajennense* is very eclectic in its feeding preferences. Adult specimens mainly infest large wild and domestic mammals, ungulates in particular [41]; immature stages are less specific in their host choice and can feed on a broader range of vertebrates, including marsupials, small and large mammals, humans, and sometimes birds [42-45]. Therefore, this tick not only can find suitable hosts practically everywhere, but it can easily be carried over large distances on wild ungulates, cattle displaced by humans, or on birds. Hillburn and Sattler [46] postulated that populations of livestock ticks, when not host-specific and in the presence of abundant host fauna, should closely approach panmixia.

Hence, two opposite evolutionary hypotheses can let us expect either that isolation mechanisms have resulted in the subdivision of *A. cajennense* into genetically distinct populations, or that persistent gene flow, maintained by dispersal on hosts, has created a genetic gradient of progressive differentiation throughout the tick distribution range. In the present study, we investigated the phylogeny and population structure of *A. cajennense* throughout its distribution range by analyzing three mitochondrial and one nuclear gene sequences. Data were used to determine the extent of genetic structure within the taxon and its compatibility with the hypothesized occurrence of distinct cryptic species, to evaluate the demographic and phylogeographical history of the tick, and to tentatively date lineage radiation events.

## Methods

### Sampling

Our samples included wild caught ticks and ticks from colonies (three Brazilian and one Colombian) (Table 1). The Brazilian colonies originated from São Paulo, Rio de Janeiro, and Rondonia and the Colombian from Cundinamarca. As colony ticks can evolve and differentiate very rapidly from wild caught specimens [47], all the colony ticks used in this study were first generation specimens that had not yet experienced the effects of inbreeding. Fresh wild samples were collected in Mexico, Argentina, Costa Rica, Perú, French Guiana, Colombia, and Ecuador. Additional ticks were selected from the holdings in the U.S. National Tick Collection (USNTC, Georgia Southern University), the Collection of the Instituto Nacional de Tecnología Agropecuaria (INTA, Rafaela, Argentina), the Coleção Nacional de Carrapatos (CNC, Faculty of Veterinary Medicine and Zootechnoloy, Univeristy of São Paulo, Brazil), and the Coleção Acarológica do Instituto Butantan (IBSP, Butantan Institute, São Paulo, Brazil). Sequences of *Amblyomma imitator* Kohls, 1958, a North American species often confused with *A. cajennense*, and *Amblyomma americanum* L. were used for outgroup comparisons.

**Table 1:** Samples used in this study

**Country**	**Code**	**Locality**	**Host**	**USNTC RML/INTA**	**Latitude**	**Longitude**
				**accession number**		
Argentina	amcaj-CHS	Salta, Dragones	*Bos taurus*	INTA 1954	23°04’S	63°20’W
Argentina	amcaj-CHO	Salta, Tala Yaco	*Equus caballus*	INTA 1957	25°52’S	64°47’W
Argentina	amcaj-YU	Salta, National Park El Rey	*Sus scrofa*	INTA 509	24°41’S	64°36’W
Paraguay	amcaj-PA	Boquerón, Estancia Faro Moro	*Puma concolor*	RML123654	21°41’S	60°01’W
Paraguay	amcaj-PA	Boquerón, Trans Chaco Road, km 580	*Mazama* sp.	RML105915	21°10’S	61°40’W
Venezuela	amcaj-VE	Bolivar, El Manteco	*Hydrochoerus hydrochoeris*	RML47831	07°08’N	61°59’W
French Guiana	amcaj-FG	Kaw Mountain	*Sus scrofa*	RML124017	04°33’N	52°09’W
French Guiana	amcaj-FG	Cayenne, Montabò Trail	Vegetation	RML124079-80	04°56’N	52°18’W
Colombia	amcaj-CO	Cundinamarca, Villeta	Vegetation	RML124466	05°01’N	74°28’W
Colombia	amcaj-CO	Cundinamarca, Villeta	Colony	RML124454	05°01’N	74°28’W
Ecuador	amcaj-EC	Pichincha, Puerto Quito	Vegetation	RML124071	00°06’N	79°16’W
Ecuador	amcaj-EC	Manabi, Banchal	*Equus caballus*	RML99809	01°37’S	80°21’W
Costa Rica	amcaj-CR	Guanacaste, Santa Rosa	Vegetation	RML124630-124639	10°50’N	85°36’W
Brazil	amcaj-RO	Rondonia, Gov. J. Teixera county	Colony I	n/a	10°38’S	63°29’W
Brazil	amcaj-AF	São Paulo, Pirassununga county	Colony II	n/a	21°59’S	47°59’W
Brazil	amcaj-AF	Rio de Janeiro, Seropedica county	Colony III	n/a	22°44’S	43°43’W
Brazil	amcaj-MG	Mato Grosso do Sul, Corumbá	*Bubalus arne*	RML122954	18°52’S	56°16’W
Brazil	amcaj-MG	Mato Grosso do Sul, Corumbá	*Sus scrofa*	RML122965	18°52’S	56°16’W
Mexico	amcaj-MX	Veracruz, Sant Andrés Tuxtla	Vegetation	RML124528	18°35’N	95°04’W
Perú	amcaj-PE	Cajamarca, Jaen, Bellavista	Cervidae (unknown species)	RML124074-76-76	05°42’S	78°48’W
U.S.A.	amcaj-TX	Texas, mixed unknown localities	Unknown	RML124534	Unknown	Unknown
U.S.A.	amimi	Texas, mixed unknown localities	Unknown	RML124015 and RML124534	Unknown	Unknown
U.S.A.	amame	Georgia, Bulloch Co., Statesboro	Vegetation	RML123785	32°25’N	81°46’W

List of localities, species-codes associated with each locality, host associations, and collection accession numbers for the analyzed samples (*A. cajennense* = amcaj; *A. imitator* = amimi; *A. americanum* = amame; CHS = Chaco Serrano; CHO = Chaco Occidental; YU = Yungas; PA = Paraguay; VE = Venezuela; FG = French Guiana; CO= Colombia: EC = Ecuador; CR = Costa Rica; RO = Brazil, Rondonia; AF = Brazil, Atlantic Forest; MG = Brazil, Mato Grosso; MX = Mexico; PE = Perú; U.S.A. = United States of America; TX = Texas). INTA numbers correspond to accesssion numbers in the collection of the Instituto Nacional de Tecnología Agropecuaria, Estación Experimental Agropecuaria, Rafaela, Argentina; RML numbers correspond to accession numbers in the United States National Tick Collection, Institute for Costal Plain Science, Georgia Southern University, Statesboro, GA.

### DNA extraction, PCR, cloning, and sequencing

DNA was extracted and the cuticles of the ticks were preserved for further morphological analysis following previously published protocols [48,49]. This involved cutting off a small portion of the postero-lateral idiosoma of each tick with a disposable scalpel, before an overnight incubation in 180 *μ*l Qiagen ATL lysis buffer (Qiagen, Valencia, CA) and 40 *μ*l of 14.3 mg/ml proteinase K (Roche Applied Sciences, Indianapolis, IN). After complete lysis of the tick tissues and repeated vortexing, the cuticle was stored in 70% ethanol and kept as a voucher specimen. The lysed tissues were further processed as previously described [48,49]. Two mitochondrial gene sequences, 12SrDNA and the control region or d-loop (DL) were amplified as previously reported [48,49]. Primers F2LITS2 = 5’ - tgagggtcggatcayatatca -3’ and McLn = 5’ - gtgaattctatgcttaaattcagggggt - 3’ [50] were used to amplify approx. 950 bp long fragments of the Internal Transcribed Spacer 2 (ITS2) [49]. Primers for the COII gene fragment, COIIF (5’-tca gaa cay wcy tty aat caa aat -3’) and COIIR2 (5’-cca caa att tct gaa cat tgw cca-3’), were selected within the cytochrome c oxidase subunit I and at the end of the COII sequence respectively, by comparing the complete mitochondrial genomes of *Rhipicephlaus sanguineus* (GenBank accession number: NC002074.1) and *Ixodes hexagonus* (GenBank accession number: NC002010.1). A touch-down annealing sequence from 55 to 46°C was used to amplfy COII sequences. PCR was performed by using a MasterTaq kit (5-Prime, Gaithersburg, MD). Each reaction contained 2.5 *μ*l of tick DNA, 2.5 *μ*l of 10 × Taq buffer, 5 *μ*l of 5 × TaqMaster PCR Enhancer, 1.5 *μ*l of MgAc (25 mM), 0.5 *μ*l dNTP mix (10 mM each), 0.1 *μ*l of Taq polymerase (5U/ *μ*l), 1.25 *μ*l of each primer from a 10 pmoles/ *μ*l stock solution (Invitrogen, Life Technologies Corporation, Grand Island, NY), and 14.6 *μ*l molecular biology grade H_2_O. The two DNA strands of each amplicon were purified and sequenced at the High-Throughput Genomics Unit (HTGU, University of Washington, Seattle, WA) and were assembled with Sequencer 4.5 (Gene Codes Corporation, Ann Arbor, MI). Because the ITS2 region occurs in multiple copies in the same organism, it was necessary to compare its variability within single tick specimens, within populations, and between populations to ensure that, in *A. cajennense*, its evolution reflected concerted evolution. For a subset of geographical regions, 2 specimens were randomly selected and the corresponding ITS2 PCR product cloned using TOPO-TA (Invitrogen). For each cloning, eight colonies containing the tick DNA insert were grown overnight in LB medium supplemented with 10% glycerol and ampicillin (Teknova, Hollister, CA), frozen, and sent for sequencing to the HTGU.

### Genetic structure and demography

Relationships between sequences were first investigated by generating unrooted networks with the statistical parsimony method implemented in TCS 1.13 [51] and a confidence interval of 95%. Haplotypes were considered to be distinct when they differed by at least one bp. Individual sequence alignments were imported into DnaSP (DNA Sequence Polymorphism) [52] version 5.10.01 for analysis of nucleotide polymorphism. The program was used to calculate haplotype and nucleotide diversity (Hd ±*SD* and *π*±*SD*) and to perform Ramos-Onsin and Roza’s R2 [53] and Fu’s Fs [54,55] neutrality tests, considered to be the most robust methods for studying the effect of demographical events on DNA sequence data [53]. For each dataset, genetic differentiation (*F*_*ST*_) between the clades identified through TCS and phylogenetic analyses were estimated by also using DnaSP. The statistical significances of all tests performed with DnaSP were estimated by the coalescent method with 95% confidence interval and 10,000 permutations. The significance of fixation indices was determined by comparison to a null distribution of these values based on 10,000 random permutations [56].

### Phylogenetic analysis

Sequences were manually aligned with McClade 4.07 OSX [57]. Secondary structure was considered in aligning 12SrDNA [48] and DL sequences, and codon organization in aligning the COII data set. The homogeneity of base frequencies across our sample was evaluated with a *χ*^2^ goodness-of-fit test using PAUP 4.0b10 [58] prior to all phylogenetic analyses. Substitution saturation was evaluated with DAMBE 5.2.0.12 [59-61]. Each data set was analyzed by maximum parsimony (MP) and maximum likelihood (ML) with PAUP, and Bayesian analysis (BA) with MrBayes 2.01 [62]. Branch support was assessed by bootstrap analysis (1000 replica) with PAUP for MP, with PHYML (100 replica) [63] in Phylogeny.fr [64] for ML, and by posterior probability with MrBayes. MP heuristic searches were performed by branch-swapping using the tree bisection-reconnection (TBR) algorithm, ACCTRAN character optimization, with all substitutions given equal weight, and with 10 random sequence addition replicates. Gaps were treated either as a 5th (in 12srDNA and D-loop analyses) or as a missing character. Maximum likelihood heuristic searches were run after the nucleotide substitution model best fitting the data was selected by Modeltest v3.7 [65]. The MP tree with the best ML score was used as the starting tree for ML searches. Two runs, with four chains each, were run simultaneously for BA analyses (1,000,000 generations). Trees were sampled every 100 iteration. Trees saved before the average standard deviation of split fragments converged to a value < 0.01 were discarded from the final sample. When necessary, the number of generations was increased so that the number of discarded samples would not exceed 25% of the total sampled trees. The 50% majority-rule consensus tree of the remaining trees was inferred and posterior probabilities recorded for each branch. The four data matrices were compared for congruence by using the partition-homogeneity test, with 100 replicates and significance threshold value P < 0.05 as implemented in PAUP [66]. Congruent data sets were combined for total evidence analyses. Two concatenated data sets, one including only mitochondrial (mtDNA) and one including both, mitochondrial a nuclear sequences (n+mtDNA), were analyzed following the same procedure outlined for the separate analyses. BA concatenated analyses were partitioned by gene and codon position (for the COII portion of the dataset).

### Molecular clock and divergence dates

In order to test substitution rate variation among lineages, for each dataset (four separate genes, mtDNA, and n+mtDNA), relative-rate tests were applied to all sister clades by using DAMBE [59]. In addition, the molecular clock hypothesis was tested by likelihood ratio-test also in DAMBE [59]. Tentative estimates of divergence time were performed by using the n+mtDNA concatenated data matrix with the relaxed molecular clock model implemented in BEAST v.1.7.4 [67,68]. BEAST analyses were run analyzing the four-gene set, but allowing for independent model parameters for each partition. The ingroup was defined as being monophyletic in agreement with the phylogenetic analysis results. The tree priors was set to Yule Process, and the molecular clock set to uncorrelated lognormal distribution. Chain lengths were set to 1,500,000 and data were sampled every 750 iterations with a random starting tree. Two independent runs were combined with LogCombiner v.1.7.4 in order to reach stable posterior distributions in Tracer v.1.5. After deleting 10% of the generated trees, the remaining trees were summarized as a combined maximum clade credibility tree by using TreeAnnotator v.1.7.4. FigTree v.1.3.1 was used to visualize tree structure, with mean divergence times. Because dated fossil records are missing for this group of ticks, node dating was based on known vicariant events which shaped the territory occupied by *A. cajennense*. Different and independent node dating strategies were applied in BEAST analysis and the results compared. In order to account for the uncertainty of these dates, normally distributed tree priors were used. Assuming a vicariance model, dating was first attempted by calibrating single nodes which is allowed when applying relaxed molecular clocks. As the basal lineages always included the PE branch, which is located in a SDTF hot spot supposedly isolated for at least 10 Mya in the Inter-Andean Valley, we surmised that the differentiation within *A. cajennense* initiated before that, most likely during a period coinciding with the beginning of the Andean uplift. Therefore, the node between out- and ingroup was set at 20 Mya ±5 Mya; the PE - AR node was set to a median of 10 Mya ±2 Mya; the origin of the NE-CO-NW clade was set to a median of 6 Mya ±1 Mya which corresponds to the end of the uplift of the eastern Cordillera in its northernmost portion; the origin of the NW clades was set at 3 ± 0.5 Mya which would represent the closure of the Isthmus of Panama. Next, we applied two different double node calibrations: the first with outgroup - ingroup at 20 Mya ±5 Mya and PE-AR at 10 Mya ±2 Mya, the second with PE-AR at 10 Mya ±2 Mya and NW-NE-CO at 6 Mya ±1 Mya.

## Results and discussion

### Taxonomic sampling

The tick sample (Table 1) contained specimens from most of the New World biomes [69] inhabited by *A. cajennense*[1]. The localities included areas with temperate grasslands, tropical and subtropical grasslands, tropical and subtropical moist broadleaf forests, tropical and subtropical dry broadleaf forests, and desert and xeric shrubland. Our sampling extended the known geographical distribution of the species to the montane shrubland of the inter-Andean Valley of Perú and the coastal mixed forest of Ecuador [1,2]. Overall, the analysis included specimens from 19 localities and 11 countries (Table 1). Collection sites or colony origins are represented in Figure 2A. For clarity, following abbreviations are used throughout the paper and in the illustrations: TX = Texas, MX = Mexico, CR = Costa Rica, EC = Ecuador, VE = Venezuela, FG = French Guiana, CHO = Chaco Occidental (Argentina), CHS = Chaco Serrano (Argentina), YU = Yungas (Argentina), AF = Atlantic Forest (Brazil), RO = Rondonia (Brazil), MG = Mato Grosso (Brazil), PA = Paraguay, PE = Perú, CO = Colombia. The phylogenetic clades were named after the general geographical range they occupy within the distribution of *A. cajennense*: NW = north-western clade (Texas, Mexico, Costa Rica, Ecuador); CO = Colombia; NE = north-eastern clade (French Guiana, Brazil-Rondonia); PE = Perú (Inter-Andean valley); EA = eastern clade (Brazil - Atlantic Forest - Mato Grosso and Argentina - Yungas); AR = Argentine clade (Argentine and Paraguayan Chaco).

### DNA extraction, PCR, cloning, and sequencing

The number of sequences obtained for each geographical area was variable due to the fact that some tick samples had been freshly collected, whereas older ethanol-preserved samples from the USNTC sometimes yielded little or no DNA at all. The amplification success also varied depending on primer sets, with 12SrDNA being the easiest gene fragment to amplify. Haplotype distributions for each gene are listed in Additional files 1, 2, 3 and 4. GenBank accession numbers are listed in the Availability of supporting data section.

### Sequence diversity and TCS analyses

The 123 12SrDNA sequences were represented by 33 distinct haplotypes, the 60 COII sequences by 26 haplotypes, and the 110 DL sequences by 31 haplotypes (Additional files 1, 2 and 3). The initial 444 bp long DL alignment needed to be reduced to 394 characters, because it included a hypervariable region alignable within clades, but not between clades. This portion of the alignment was, therefore, eliminated from further data analysis. The TCS analyses (95% parsimony cut-off level) separated mitochondrial haplotypes in distinct networks that could not be joined even after reducing the cut-off level to 70–80% (Additional files 5, 6 and 7). Independently on the gene analyzed, the geographical composition of the subnetworks (labeled NW, CO, NE, EA, AR, and PE) was the same. NW consistently included samples from TX, MX, CR and EC; NE samples from FG and RO; CO samples from Colombia; EA samples from YU, MG, AF; AR samples from CHO, CHS, and PA; and PE samples from the inter-Andean Valley of Perú. The network subdivision indicated that mitochondrial lineages of *A. cajennense* were deeply split and separated by a large number of parsimonious steps. It also showed that the three mitochondrial markers were congruent in splitting *A. cajennense* in the exact same subunits. In general, haplotypes were mostly unique to their area of origin, with the exception of haplotypes consistently shared by EC, MX, and CR, and by AF, YU, and MG. In addition, when available, the PA haplotypes were identical or at least very similar (1 bp difference) to the CHO, rather than to the CHS haplotypes, which is geographically easily explained. Also, when available, the VE and FG haplotypes clustered together. The 78 ITS2 gene sequences were collapsed into 20 distinct genotypes (Additional file 4). The ITS2 sequences were more conserved than the mitochondrial gene sequences with, for instance, French Guiana and Rondonia sharing identical genotypes. Similarly, although, separated by long branches (10 steps), CO and NE were included in the same network as were NW and EA (Additional file 8). By increasing the cut-off level to 97% (approx. 24 bp/825 total bp), TCS resulted in the exact same split recorded for the mitochondrial genes. The individual networks were structured, often including long branches and loops, and lacked the star patterns identifying recent sudden radiations. The populations they represented, therefore, appeared to have diversified over time. These findings indicated that the analysis of both mitochondrial and nuclear markers revealed congruent marked divergence between samples from different areas. The lack of connection between subnetworks did not allow us to determine the possible geographical pathways followed by A. cajennense throughout its radiation around the Amazon basin. The ITS2 network, however, indicated that the NW ticks evolved from the EA cluster, and that NE and CO were more closely related to each other than to the other groups. Measures of ML sequence divergences (Tables 2 and 3) showed that diversity within clades was very low varying from 0 to 4% (reaching 4% only with COII, the most diverse gene). Values between clades, between outgroups and ingroup, and between outgroups were comparable and typically about 8–10 times higher than intra-clade values, with a single exception: CO and NE only differed by 1.15% in ITS2, which is significantly lower than the other observed inter-clade values. This was the only significant discrepancy observed between mtDNA and ITS2 data, indicating that the separation between CO and NE may not be as clearly defined as it is between other clades when using a nuclear recombining rather than a mitochondrial marker. In general, however, our data showed that the diversity between clades was similar or higher than that observed between the two very different outgroups or between the outgroups and the ingroup. This strongly suggested that the differences between the six clades identified through TCS and phylogenetic analyses (see below) were compatible with the occurrence of six separate species.

**Table 2:** Divergence between clades

**COII/12SrDNA**	**AR**	**PE**	**EA**	**CO**	**NE**	**NW**
AR	0–0.54/**0–0.60**	15.61–17.89	16.57–20.86	19.81–21.71	**18.64–19.86**	17.75–20.48
PE	34.98–36.82	0–4.00/**0–0.88**	15.38–20.01	21.00–23.51	**18.90–20.54**	18.68–23.23
EA	27.82–30.76	43.63–47.37	0–1.10/**0–2.13**	11.14–13.18	11.43–12.92	**8.33–10.97**
CO	28.20–29.25	40.41–42.71	18.63–19.39	0.00/**0–0.29**	7.97–8.40	8.35–9.63
NE	28.42–31.47	49.39–54.60	16.72–19.61	11.78–12.66	0–1.09/**0–0.58**	7.63–9.17
NW	25.80–28.11	39.41–43.22	20.75–23.40	14.58–16.14	16.21–18.60	0–1.50/**0–1.17**
DL/**ITS2**						
AR	0–1.07/**0–0.25**	3.42–3.85	7.23–7.70	8.99–9.32	**8.74–9.06**	7.27–7.59
PE	9.49–11.46	0–0.52/**0–0.38**	7.89–8.48	9.58–9.85	**9.31–9.58**	7.63–8.18
EA	17.47–23.04	21.18–26.58	0–2.52/**0–0.88**	4.38–4.98	3.92–4.21	**1.41–2.07**
CO	10.64–11.73	13.69–14.77	13.41–15.82	0.00/**0.00**	1.15	4.11–4.41
NE	13.92–16.45	16.13–19.24	12.7116.38	5.90–6.65	0–1.32/**0.00**	3.65–3.94
NW	12.14–14.37	16.05–17.87	13.25–15.59	8.29–8.71	7.80–9.43	0–0.26/**0–0.50**
n+mtDNA/**mtDNA**						
AR	0–0.48/**0–0.64**	23.25–24.07	26.17–28.97	22.68–24.02	**24.38–26.20**	22.67–24.42
PE	11.44–11.64	0–0.19/**0–0.24**	32.19–36.59	29.76–31.15	**34.17–36.05**	29.74–31.84
EA	14.74–15.39	16.71–18.03	0–1.19/**0–1.78**	16.69–17.96	16.46–17.68	**17.08–18.31**
CO	14.36–14.52	16.81–17.09	9.82–10..41	0–0.05/**0–0.08**	9.72–10.13	11.65–12.45
NE	14.82–15.29	17.99–18.62	9.48–10.10	5.40–5.59	0–0.63/**0–1.03**	12.18–13.25
NW	13.41–13.78	15.74–16.27	8.33–8.87	7.63–8.11	7.66–8.20	0–0.38/**0–0.55**

Maximum likelihood distance values between clades (in percentage). 12SrDNA = small mitochondrial ribosmal subunit, COII = Cytochrome oxidase c subunit II, DL = control region or d-loop; ITS2 = intergenic spacer 2.12SrDNA, ITS2 and mtDNA (mitochondrial concatenated) above diagonals (in bold), COII, DL, and n+mtDNA (nuclear and mitochondrial concatenated) below diagonals. NW = Ecuador, Costa Rica, Mexico, Texas; CO = Colombia; NE = French Guiana + Venzuela + Brazil (Rondonia); EA = Brazil (Atlantic Coast, Mato Grosso) + Argentina (Yungas); AR = Agentina + Paraguay (Chaco); PE = Perú.

**Table 3:** Summary of divergence values

**ML divergence**	**Within**	**Between**	**Between**	**Between**
**values (%)**	**clades**	**clades**	**out-ingroup**	**outgroups**
Gene				
12SrDNA	0.00–2.13	7.63–23.51	18.61–34.06	9.17
COII	0.00–4.00	11.78–54.60	31.74–60.84	25.33
DL	0.00–2.52	5.90–26.58	17.86–34.01	13.25
mtDNA	0.00–1.78	9.72–36.59	30.79–42.63	18.75
ITS2	0.00–0.88	1.15–9.85	9.39–14.63	6.42
n + mtDNA	0.00–1.19	5.40–18.62	18.48–21.49	11.36

Maximum likelihood distance values at different hierarchic levels (in percentage). 12SrDNA = small mitochondrial ribosmal subunit, COII = Cytochrome oxidase c subunit II, DL = control region or d-loop, ITS2 = intergenic spacer 2, MtDNA = concatenated mitochondrial genes, n + mtDNA = concatenated nuclear and mitochondrial genes.

### Phylogenetic analyses

**Figure 3:**
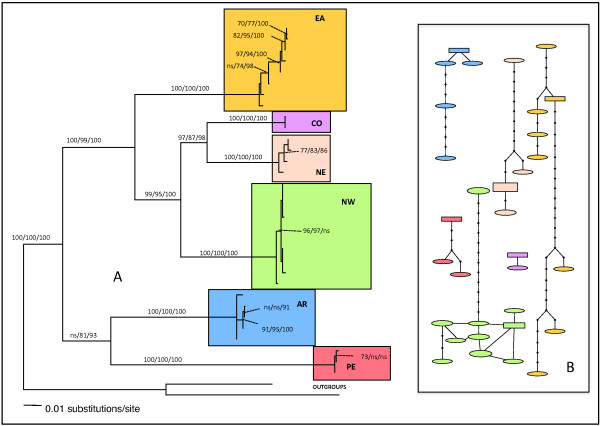
**Mitochondrial gene tree.(A)** Tree representing the relationships between *A. cajennense* inferred by ML analysis of the concatenated mitochondrial gene sequences. NW = Texas, Mexico, Costa Rica, Ecuador clade, NE = French Guiana and Rondonia (Brazil) clade, CO = Colombia, EA = Yungas Argentina + Atlantic Forest and Mato Grosso of Brazil, AR = Chaco (Argentina and Paraguay), PE = Inter-Andean Valley of Perú. Numbers over the branches represent MP bootstrap values (1000 replicates), ML bootstrap values (100 replicates), and BA posterior probabilities respectively. **(B)** Unrooted TCS Network (95% parsimony cut-off). Same colors in A and B represent the same samples.

**Figure 4:**
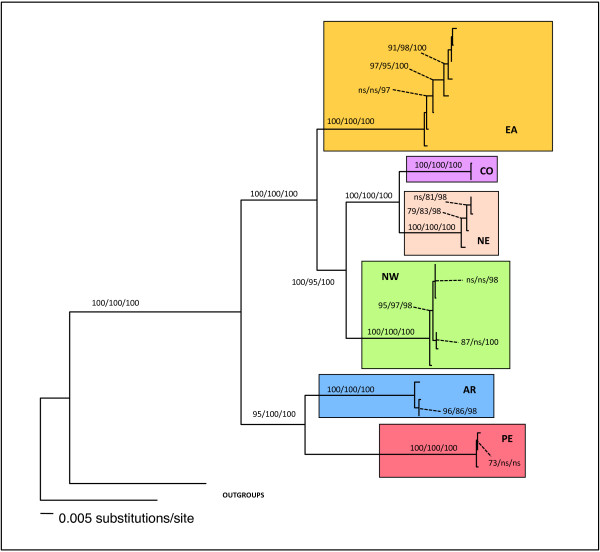
**Total evidence analysis tree.** Tree representing the relationships between *A. cajennense* inferred by ML analysis of the concatenated nuclear and mitochondrial gene sequences. NW = Texas, Mexico, Costa Rica, Ecuador clade, NE = French Guiana and Rondonia (Brazil) clade, CO = Colombia, EA = Yungas Argentina + Atlantic Forest of Brazil, AR = Chaco (Argentina and Paraguay), PE = inter-Andean Valley of Perú. Numbers over the branches represent MP bootstrap values (1000 replicates), ML bootstrap values (100 replicates), and BA posterior probabilities respectively.

Tree properties and scores are shown in Table 4 and substitution models selected by the Akaike Information Criterion in Modeltest [65] for each alignment in Table 5. Phylogenetic reconstructions obtained with the separate datasets are shown in Additional files 5, 6, 7 and 8, whereas the mtDNA and the n+mtDNA trees are shown in Figures 3 and 4, respectively.

**Table 4:** Tree scores

**MP trees**	**Alignment**	**PI**	**MP number of**	**MP tree**	**CI**	**RI**	**RC**	**Gap**	**MB**	**ML -lnL**	**MB -lnL**
	**length**		**saved trees/**	**length**				**treatment**	**generations**	**score**	**score**
			**of islands**								
12SrDNA	354	96	216/1	114	0.77	0.94	0.72	5th	1,500,000	1337.06	1366.07
COII	561	170	42	378	0.69	0.89	0.61	Missing	1,000,000	2460.08	2460.1
D-loop	394	85	233/1	210	0.72	0.916	0.66	5th	1,000,000	1512.72	1515.07
ITS2	825	129	10	230	0.9	0.95	0.86	5th	1,000,000	2206.71	2206.71
mtDNA	1310	352	3	730	0.71	0.92	0.66	Missing	1,000,000	5184.39	5184.39
n+mtDNA	2137	464	3	925	0.75	0.91	0.68	Missing	1,000,000	7174.75	7714.74

Trees scores obtained by maximum parsimony (MP), maximum likelihood (ML), and Bayesian (MB) analyses for each of the datasets; PI= parsimony informative characters, CI = consistency index, RI = retention index, RC = rescaled consistency index. 12SrDNA = small mitochondrial ribosmal subunit, COII = cytochrome oxidase c subunit II, DL = control region or d-loop, ITS2 = intergenic spacer 2, mtDNA = concatenated mitochondrial genes, n + mtDNA = concatenated nuclear and mitochondrial genes.

**Table 5:** Substitution models

**Gene**	**Model**	**Ti/Tv**	**A > C**	**A > G**	**A > T**	**C > G**	**C > T**	**G > T**	***α***	**I**	**% A**	**% C**	**% G**	**% T**
12SrDNA	GTR+I		0.33	7.03	3.01	0	11.93	1	Equal	0.62	41.7	8.7	14.5	35.1
COII	TRN+G		1	5.75	1	1	8.95	1	0.26	0	37.8	17.9	7.9	36.4
D-loop	HKY+I+G	1.04							1.07	0.57	30.9	13.9	13.0	42.2
ITS2	GTR+I		1.3	5.05	1.32	0.22	0.88	1	Equal	0.56	14.7	37.9	28.9	18.5
mtDNA	GTR+G		0.76	4.29	1.53	0.57	7.58	1	0.21	0	37.0	13.8	11.4	37.8
n+mtDNA	TVM+I+G		1	4.76	2.88	0.43	4.76	1	1.03	0.49	27.6	24.0	18.2	30.2

Substitution models selected by the Akaike Information Criterion in Modeltest for each dataset, with substitution matrices (x >y indicating mutation from nucleotide x to nucleotide y), Ti/Tv = transition/transversion rates, *α* = shape of gamma distribution, I = proportion of invariable sites, and base frequencies (A, C, G, T) in percentage. GTR + I = General Time Reversible + Invariable model; GTR + Gamma = General Time Reversible + Gamma model; TRN + G = Tamura Nei + Gamma model; HKY + I + G = Hasegawa-Kishino-Yano + Invariable + Gamma model; TVM + I + G = Transition Model + Invariable + Gamma model.

#### Mitochondrial genes

With very few exceptions, for each gene, the three phylogenetic methods (MP, ML, and BA) generated trees with overall similar topologies and lineage support. Although phylogenetic reconstructions based on the three mitochondrial genes were not topologically fully congruent (Additional files 5, 6 and 7), they consistently identified the same monophyletic units which corresponded to the subnetworks identified through TCS analysis. In general, 12SrDNA sequences appeared to provide better overall resolution than COII or DL, confirming the good level of information offered by small ribosomal subunit genes when investigating relationships among closely related taxa, but also within a single species [48,49,70,71]. DAMBE did not reveal significant saturation in the 12SrDNA dataset. In the 12SrDNA reconstruction, PE was the basal well-supported lineage followed, in order of divergence, by the strongly supported AR and EA clades. The most recently evolving lineage grouped CO-NE-NW, which were each monophyletic. NE and CO were sister clades. For the COII dataset, saturation levels were tested for the whole data matrix, for each codon position separately, and for the two first codon positions together. None of these sets showed significant nucleotide saturation based on transition and transversion rates. Therefore, third codon positions were included in all further analyses. The topology of the COII tree differed from the 12SrDNA tree in few main aspects: PE-AR clustered in a monophyletic lineage, the lineages grouping the remaining clades were only supported in BA, and the position of CO was unresolved. The DL is one of the most variable regions in the mitochondrial genome [72]. Although it’s usefulness for the study of the evolutionary history of arthropods has not yet been thoroughly investigated, this gene has proved to be informative at the intraspecific level for some arthropods [73,74] and for two tick species, *Ixodes ricinus* and *A. variegatum*[49,70]. In this study, the overall DL tree structure also lacked in support, particularly at the base of the otherwise well-supported CO-NE-EA-NW split. Although substitution saturation was initially suspected to be at the origin of the lack of resolution in DL and COII, all tests failed to find significant saturation in the mitochondrial markers.

#### ITS2 sequences

Mitochondrial and nuclear genes do not always portray the same evolutionary history due to their distinct inheriting mechanisms. The non-recombining mitochondrial genes usually provide excellent phylogeographical information. Although they are increasingly being used for the delimitation of species (DNA barcoding), the identification of mitochondrial deep divergent splits may merely reflect past biogeographical events that do not always imply speciation. On that account, and in order to verify whether or not the analysis of a nuclear gene would result in the same clear cut subdivision of our samples, sequences of the noncoding rapidly evolving ITS2 regions were also sequenced. However, nuclear ribosomal DNA (rDNA) is known to occur in multiple copies in the genome. Different copies within the same individual usually evolve as a single-copy gene through concerted evolution [75,76]. Nevertheless, exceptions to this rule have been observed in arthropods including ticks, particularly in the noncoding rDNA ITS regions which separate the transcribed genes [77-82]. If ribosomal DNA copies within a specimen are more diverse than between specimens, phylogenetic analyses using one representative sequence for each sampled specimen may not accurately represent relationships by descent. Phylogenetic and population genetics studies in ticks have, however, paradoxically often been based on analyses of ITS sequences [50,83-88]. The ITS2 gene proved to be an informative marker at the intrageneric level in Neotropical *Amblyomma* species [87], and within populations of *Amblyomma americanum*[47], but not within *Amblyomma variegatum*[49], probably a more recently evolving species. After sequencing randomly chosen cloned ITS2 sequences from single specimens, the variability within (0.00–0.15%) was slightly lower than that between specimens from the same clade (0.00–0.88%). Moreover, when the intra-specimen substitutions were visually inspected, it became clear that they occurred as parsimony uninformative singletons and never involved informative segregating sites. Consequently, we concluded that ITS2 gene fragments were suitable markers for evolutionary studies in *A. cajennense*. The ITS2 tree topology (Additional file 8) was strongly supported at each hierarchical level and identified the same monophyletic clades revealed by the analysis of the mitochondrial genes. PE and AR were clustered in the basal monophyletic clade, a sister group to the well-supported EA-NW-NE-CO lineage. Within the latter, EA-NW, and NE-CO were sister branches.

#### Concatenated datasets

The four data matrices proved to be phylogenetically informative with relatively little homoplasy (Table 4), with homogeneous base frequencies, and non significant substitution saturation. Partition homogeneity tests revealed that 12SrDNA-DL and COII-DL were congruent datasets, with *p* = 0.86 and *p* = 0.74 respectively, while 12SrDNA-COII were not (*p*=0.04). Because the latter significance level was low, and there was no significant conflict between DL and both other mitochondrial genes, the three datasets were concatenated. There was also no significant conflict’s signal between mtDNA and ITS2 (*p*=0.28) and, consequently, the four datasets were combined for total evidence analysis. The topology of the two concatenated trees (Figures 3 and 4) was identical, with strongly supported nodes (> 90%) at all levels, if we exclude the AR-PE lineage which was less supported in the MP mtDNA tree. The six main clades were all characterized by 100% bootstrap support. In order of divergence from basal to crown, the AR-PE clade was followed by the EA, the NW, and the NE-CO clades. The nodes giving rise to the six clades were set deep in the phylogenies, while the terminal branches were often polytomic (with the exception of the more diversified EA clade).

### Historical demography and population genetics

We used diversity statistics and neutrality tests to look for signals of population growth in each of the clades (Table 6). Under a model of population expansion, because newer haplotypes are retained in the population and are recently derived, Hd is expected to be high whereas nucleotide diversity is expected to be low. It must be noted that, because indels are excluded from the DnaSP analyses, the number of haplotypes detected by DnaSP is lower than the number of haplotypes listed in Additional files 1, 2, 3 and 4. Although not all our samples included sufficient specimens or different haplotypes for robust analysis, in the total sample, observed haplotype diversity was significantly higher than expected (Table 6). Nucleotide diversity varied among mitochondrial genes, but was not significantly lower than expected in the mtDNA and the nuclear datasets within clades and in the total sample. This is an indication that the six clades constitute stable distinct populations and are not the result of recent population expansion or growth. Fu’s F and Ramos-Onsins R_2_ statistics performed on the nuclear and mtDNA datasets were not significant (Table 6), corroborating the idea that the six clades did not experience any sudden demographic event and that they have been evolving in stable conditions for a long period. The important level of genetic diversity observed in the clades may indicate that the ancestral taxon was genetically diverse before it became fragmented and that this genetic diversity was maintained in isolated allopatric refugia because they provided stable environmental conditions allowing for genetic accumulation. Relatively important genetic diversity can also be the result of limited dispersal and recent evidence [89] strongly corroborates the hypothesis [24] that many ticks are more strictly dependent on suitable environmental conditions than on the availability of specific hosts. Therefore, even if the tick theoretically could feed on a number of vertebrate hosts with high dispersal ability, it appears that they would not venture too far from the ecological niche they prefer. Whether some of this diversity is maintained through secondary contact along the suture zones between the areas occupied by the six clades will have to be determined through intensive sampling efforts and additional molecular analyses. In terms of population structure, F _*ST*_ values between clades for each gene separately and for the concatenated datasets were very high (i.e. 0.91 – 0.99 for 12SrDNA, 0.92 – 1.00 for DL, 0.96 – 1.00 for COII, 0.80 – 1.00 for ITS2, and 0.95 – 0.99 for mtDNA) and were all highly significant with *p* values < 0.001 and < 0.01 confirming, once more, that the six lineages are genetically very distinct form each other.

**Table 6:** DNA polymorphism statistics and neutrality tests

	**Clade**	**NW**	**CO**	**NE**	**EA**	**AR**	**PE**	**Total**
**12SrDNA**	Sample size/H	51/9	7/2	16/2	15/6	10/3	24/4	123/24
	Hd ± SD	0.58 ± 0.07	0.48 ± 0.17	0.23 ± 0.13	0.85 ± 0.06	0.64 ± 0.10	0.49 ± 0.11	0.83 ± 0.03
	*π*(%) ± SD	0.24 ± 0.04	0.14 ± 0.05	0.14 ± 0.07	1.2 ± 0.15	0.22 ± 0.05	0.16 ± 0.06	7.6 ± 0.3
**DL**	Sample size/H	46/2	6/1	14/5	15/10	10/4	19/3	110/25
	Hd ± SD	0.08 ± 0.05	0	0.50 ± 0.16	0.91 ± 0.06	0.73 ± 0.10	0.20 ± 0.12	0.81 ± 0.03
	*π*(%) ± SD	0.02 ± 0.00	0	0.59 ± 0.17	0.95 ± 0.13	0.47 ± 0.46	0.05 ± 0.03	7.0.4 ± 0.33
**COII**	Sample size/H	24//4	3/1	12/6	11/7	5/5	5/3	60/25
	Hd ± SD	0.57 ± 0.09	0	0.75 ± 0.12	0.91 ± 0.07	1 ± 0.13	0.70 ± 0.22	0.92 ± 0.02
	*π*(%) ± SD	0.39 ± 0.09	0	0.33 ± 0.10	0.60 ± 0.62	0.36 ± 0.07	1.44 ± 0.80	9.95 ± 0.63
**ITS2**	Sample size/H	20/5	7/1	15/1	18/5	5/3	9/4	74/19
	Hd ± SD	0.44 ± 0.13	0	0	0.77 ± 0.05	0.70 ± 0.22	0.75 ± 0.11	0.90 ± 0.02
	*π*(%) ± SD	0.09 ± 0.03	0	0	0.24 ± 0.05	0.10 ± 0.04	0.12 ± 0.03	3.60 ± 0.27
	Fu’s F	-1.912	-	-	0.39	-0.829	-1.039	14.341
	R2	0.1074	-	-	0.1439	0.2449	0.1667	0.1461
**mtDNA total**	Sample size/H	17/6	2/2	9/4	8/8	5/5	3/3	44/27***
	Hd ± SD	0.82 ± 0.06	1.00 ± 0.25	0.69 ± 0.15	1.00 ± 0.06	1.00 ± 0.13	1.00 ± 0.27	0.94 ± 0.02***
	*π*(%) ± SD	0.22 ± 0.05	0.08 ± 0.04	0.32 ± 0.13	0.84 ± 0.15	0.36 ± 0.09	0.21 ± 0.06	8.4 ± 0.61
	Fu’s F	0.347	-	2.112	-2.011	-1.481	-	10.292
	R2	0.1324	-	0.1797	0.1583	0.2111	-	0.1626

Sample size and diversity measures for each gene and for each clade. Hd = haplotype diversity, Gd = gene diversity, H = number of haplotypes or genotypes, *π* = nucleotide diversity in %, SD = standard deviation. Results of Rozas-Onsins’s (R2) and Fu’s F neutrality tests were recorded for the combined mitochondrial and the nuclear datasets. NW = Ecuador, Costa Rica, Mexico, Texas; CO = Colombia; NE = French Guiana + Venzuela + Brazil (Rondonia); EA = Brazil (Atlantic Coast, Mato Grosso) + Argentina (Yungas); AR = Agentina + Paraguay (Chaco); PE = Perú. *** = *p* < 0.001. 12SrDNA = small mitochondrial ribosmal subunit, COII = cytochrome oxidase c subunit II, DL = control region or d-loop, ITS2 = intergenic spacer 2, mtDNA = concatenated mitochondrial genes.

### Molecular clock and divergence dates

**Figure 5:**
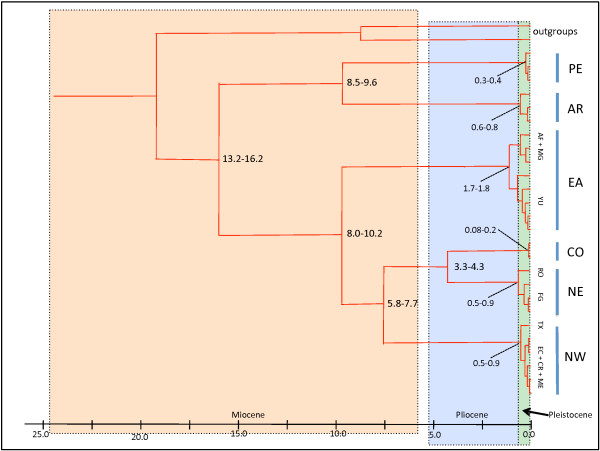
**Node dating.** Tentative dating of divergence time of the main nodes in the concatenated nuclear and mitochondrial gene tree. The two values on each node correspond to the estimates obtained by using two different calibration strategies (see Table 7).

**Figure 6:**
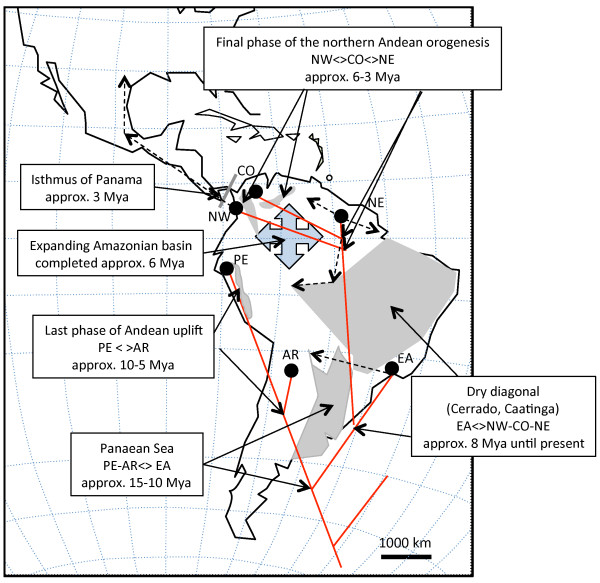
**Geographical barriers and chronology.** Biogeographical events which may have shaped diversification within *A. cajennense* and their approximate chronology. Solid red lines represent Miocene phylogenetic lineages (not to scale). Dashed lines represent dispersal and differentiation which took place either at the end of the Pliocene or during the Pleistocene.

With all data sets, relative rate tests did not reveal significant differences between sister taxa at all evolutionary hierarchic levels within each tree. Rates were also not statistically different between ingroup and outgroup sequences, indicating that rate variation among lineages would not be the cause of misleading divergence date estimations. The molecular clock hypothesis was tested for each gene by the least-square method and the likelihood ratio test implemented in DAMBE [59]. The molecular clock hypothesis could not be rejected in any of them with the exception of the LRT in ITS2 (*p*=0.03). As this was the only significant finding and its level of significance was low, we deduced that, overall, the datasets did not depart significantly form clockwise evolution. Divergence dates obtained by the different calibration criteria on the total evidence dataset (one node vs. two nodes) resulted in similar average radiation times, although the confidence intervals observed with the one-node calibrations were approximately 10% more important. The average dates obtained with the two two-node calibrations are shown in Figure 5 and the corresponding confidence intervals are listed in Table 7. If the confidence intervals were quite large at the base of the tree, particularly between outgroups and ingroup, they became, however, less prominent towards the crown bifurcations. Nevertheless, the average dates inferred with the two calibrating criteria were well within confidence intervals of both analyses. Therefore, independently on the calibration strategy applied to the analysis and within the hypothesized timeframe, the timing of the diverging events appeared to be reasonably consistent. Naturally, node dating based solely on what we believe are the biogeographical events involved in shaping the topology of the area occupied by *A. cajennense* may be misleading as the geological records are also estimates. Nevertheless, for lack of fossil records and based on these tentative evaluations, we can formulate some hypotheses about the temporal phylogeographical sequence of events based on an allopatric vicariant model of divergence (Figure 6). We can assume that the geographical distribution of the ancestor of *A. cajennense* was larger and covered the northern half of South-America during the first half of the Miocene, in environments that corresponded to the overall present ecological requirement of the tick [1]. Reasons for PE and AR being isolated in the same clade are difficult to establish. One possible explanation is that the ancestors of the AR-PE and the ancestors of the EA-NW-NC-CO clades became isolated in the south-west and north-east, respectively, of the Oceanic introgression called Paranean Sea in the middle - late Miocene [90] (Figure 6). The PE clade, like other Andean lineages [91,92], is separated by deep divergence from the related lowland taxa. We assume that PE became trapped between the progressively rising Andean Cordilleras because the area where our Peruvian samples were collected is thought to be a SDFT refugium isolated for at least 10-5 Mya since the rapid final phase of the Central Andean uplift [31,93,94]. After the retreat of the Paranean Sea from the end of the Miocene to the end of the Pliocene (10-3 Mya) [90] the AR and the EA clades may have reached each other again within a secondary contact zone corresponding to north-western Argentina. The NE-CO-NW clade split from the EA around 8-9 Mya when lacustrine ecosystems and swamps covered large parts of what would become Amazonia, creating an environment unsuitable for *A. cajennense*. Therefore, we can surmise that the tick populations were progressively being shifted to the periphery of the rain forest [35,95,96]. EA became established in the Atlantic Forest along the eastern coast of modern Brazil and in part of the Cerrado which is one of the elements of the so-called dry diagonal separating the Amazonian rain forest from the Atlantic Forest. A number of species are known to occur concomitantly in the Atlantic Forest and the Cerrado biome [97]. The fact that EA ticks are also found in forests of the Yungas in north-western Argentina is not surprising as the Amazon forest and the Atlantic Forest were reportedly linked by continuous forest [97,98]. The phylogeographical history of the NE-NW-CO clade is difficult to unravel. Nevertheless, we can speculate that it became separated from the EA clade in the late Miocene and that it adapted to ecosystems at the periphery of the Amazon basin along the western edge of the the pre-existing Cerrado-Caatinga formations. Diversification times for Cerrado woody lineages date back to 9.8 Mya [99]. The divergence between NW and NE-CO might have happened after their common ancestor had established itself along the northern coast of South America. The origin of the NW, a mostly Central and North American clade with an extension along the Pacific coast of Ecuador, seems to predate the NE-CO split (approx. 6-8 Mya) and may coincide with the end of the orogenesis of the Central Northern Cordillera which could have confined this population along its western slopes. In addition, diversification could also have been driven by the presence of the Pebas aquatic system which isolated the north-western edge of South America from the Guiana shield [100] in the early-middle Miocene (24-11 Mya). The dating, however, would not coincide with the split between NW and NE-CO, which is more recent. From there, the ticks probably reached Central and North America after the closure of the Isthmus of Panama at the beginning of the Pliocene [101], although earlier dispersal on birds over water cannot be dismissed. The NE group includes samples collected in French Guiana and Rondonia and, in order to link these disjunct areas, the most likely hypothesis would suggest that the populations belonging to this clade occupied the northern coast of South American and, following the western side of the Cerrado-Caatinga corridor, exploited relatively drier environments within the rain forest. These ecosystems developed and have persisted since the early Miocene [102] as an entity distinct from the newly formed Amazon basin. The closest relative of the NE lineage is the CO and the most parsimonious explanation for its present location would involve vicariant separation from NE in the late Miocene, when the northern part of the Eastern Cordillera completed its orogenesis. More specifically, the eastern Cordillera started developing 25 Mya but only reached appreciable elevations in the late Miocene, as did the Merida Andes in Venezuela [103,104]. The Sabana de Bogotà, where the Magdalena Valley is located and where the Colombian samples were collected, progressively increased in elevation between 15 and 3 Mya. The split between the NE and the CO lineages appears to coincide with the end of the rise of the Sabana de Bogotà. During approximately 3 to 9 Mya the main *A. cajennense* lineages did not appear to diversify indicating that they might have experienced a long period of genetic stability probably maintained by non-fluctuating environmental conditions, an hypothesis also confirmed by our demographic data. Nevertheless, we cannot exclude that extinction events during the late Miocene reduced the number of lineages to the extant ones. Diversification resumed in the late Pliocene (EA) and during the Pleistocene probably in response to the well documented climatic variability of the Quaternary age. Alternatively, as the basal lineages are PE and AR, we could imagine that the tick was well established in what corresponds now to its south-western range in the early Miocene and dispersed from there into acceptable ecological niches that developed around the newly formed Amazon Basin. The deep lineage split between clades would, however, rather suggest that the tick already occupied its present overall distribution area before the Amazon basin completed its development (6 Mya - [96]) and that its present allopatric distribution is the result of habitat fragmentation and not of a progressive northward dispersal.

**Table 7:** Tentative evaluation of divergence times

**Clade**	**Calibration 1**	**Calibration 2**
Ingroup	13.2 (8.0–18.5)	16.2 (9.8–23.5)
AR-PE	8.5 (5.5–11.9)	9.6 (6.0–13.4)
EA-NE-NW-CO	8 (4.9–11.5)	10.2 (4.9–16.5)
NW-NE-CO	5.8 (2.7–8.4)	7.7 (3.5–12.6)
NE-CO	3.3 (0.9–4.9)	4.3 (1.8–7.1)
PE	0.3 (1.1 ^-2^ – 0.8)	0.4 (1.9 ^-2^ – 1.2)
AR	0.6 (9.4 ^-2^ – 1.5)	0.8 (0.1–2.4)
EA	1.7 (0.3–7.8)	1.8 (0.3–5.5)
NE	0.5 (0.1–1.0)	0.9 (0.2–2.5)
NW	0.5 (6.2 ^-2^ – 1.1)	0.9 (0.1–3.8)
CO	8.1-2 (1.8 ^-3^ – 0.2)	0.23 (2.1 ^-3^ – 0.7)

Comparison of divergence times found in this study with calibration 1 (outgroup-ingroup at 20 ± 5 Mya and PE-AR at 10 ± 2 Mya) and calibration 2 (PE-AR at 10 ± 2 Mya and NW-NE-CO at 6 ± 1 Mya). Times are in millions of years (Mya) and refer to mean estimated ages (and confidence intervals) of the specified clade. NW = Ecuador, Costa Rica, Mexico, Texas; CO = Colombia; NE = French Guiana + Venzuela + Brazil (Rondonia); EA = Brazil (Atlantic Coast, Mato Grosso) + Argentina (Yungas); AR = Agentina + Paraguay (Chaco); PE = Perú.

## Conclusions

### Phylogenetic analyses and taxonomic considerations

Our data demonstrate that *A. cajennense* is subdivided into six genetically distinct groups. The question arises, therefore, as to whether or not these reciprocally monophyletic units represent different species. Regardless of the gene used, the six groups are characterized by unique fixed characters and mutually exclusive sequences, which is in agreement with the phylogenetic species definition [105]. The level of genetic divergence between the clades is comparable to, or much higher than, genetic diversity recorded between different *Amblyomma* species represented here by the outgroup taxa. Significant very high among-clade differentiation estimates also confirm that there is significantly little genetic exchange between these entities. These results prompted a thorough reassessment of the morphology of *A. cajennense*, which resulted in the identification of previously ignored fixed phenotypic characters, differentiating the six groups [106]. Therefore, if allopatric speciation between populations of *A. cajennense* has occurred, it is certainly not “*cryptic*”. While this study was completed, some of us [12,13] carried out cross-breeding experiments with colonies established for some of the clades. The experiments showed that colonies of AR, EA, NE and CO were not compatible, at least in laboratory conditions. Therefore, morphological and biological studies further strengthen our results and collectively argue for the occurrence of six species, indicating that Koch [3] and Tonelli-Rondelli [4,5] were mostly correct in their species delimitations. Formal descriptions of these species will be provided separately [106].

### Phylogeographical patterns and tentative dating of the major lineages splits

The presence of *Amblyomma* fossils in Dominican amber deposits, dated from the late Oligocene-early Miocene (approx. 25 Mya), indicates that the genus was already well established in the New World at the end of the Oligocene. In present times, adults *A. cajennense* appear to favor ungulate hosts, such as horses, cows, deer, and pigs, but can also be found feeding on carnivores, marsupials and Edentata. Therefore, although the present preferred hosts invaded the Neotropical region from the Northern Hemisphere after the establishment of the Panama Isthmus, we can surmise that suitable hosts were available earlier. The geographical area occupied by *A. cajennense* significantly overlaps, or is closely adjacent to, the vestigial refugia of SDTF (Figure 2A-B) which are disjunct geographical areas with similar ecological conditions, identified mostly through the study of hundreds of plant lineages with coincidental distribution patterns [14,15]. In addition, the tick distribution includes also areas of Chaco and Cerrado which were excluded by Pennington from the definition of SDTF [15] based on soil and vegetation compositions. Nevertheless Chaco and Cerrado are characterized by seasonality and long dry seasons like SDTF. Ticks, unlike plants, are more likely to depend on climatic conditions than on soil composition. Although the ecology of the ancestral SDTF was probably very similar, their long isolation from each other also coincided with gradual ecological modifications to which the ticks responded through increasing allopatric adaptation. For instance, while some clades may share similar coastal ecological conditions (EA, NE in French Guiana, and NW) there is no doubt that AR and PE occur in very different environments, the arid Chaco and the montane dry Inter-Andean valleys. The EA and AR ticks can be found 50 km apart, but they do not appear to venture out from their respective endemic areas. Nevertheless, a thorough exploration of the possible secondary contact zones between clades has yet to be undertaken and may shed some light on the real level of incompatibility in the natural environment between the identified species. Our attempt at node dating is in part speculative, as fossil records are largely missing for ticks, and because the dating of biogeographical topological modifications are also sometimes conjectural. Nevertheless, the phylogeography of *A. cajennese* is, sometimes partly but sometimes extensively congruent with the phylogeographical estimates generated for organisms with similar trans-Amazonian distributions, which include reptiles, rodents, and birds [16-20]. In some cases, divergence dating in these studies were supported by dated fossil records. Not only are the radiation patterns similar, but their dating often also matches our results, indicating that *A. cajennense* is only one of many organisms with an early Miocene origin and with affinity for trans-Amazonian regions with marked seasonality and long dry seasons [16-20]. Interestingly, the most extensive phylogeographical similarities are found in unrelated taxa, such as caviomorph rodents [21] and *Crotalus* spp. snakes [22]. To the best of our knowledge, this is the first study dealing with an invertebrate with such a large and typical trans-Amazonian distribution range. The identification of these new species is not of solely taxonomic interest. It also has important implications for public health issues, because these ticks are vectors of important human pathogens. Accordingly, the subdivision revealed by our study also appears to correlate, in some cases, with distinct tick-pathogen associations [107-111].

## Availability of supporting data

GenBank accession numbersfor 12SrDNA sequences are: EU791583-97, EU791599-600, EU791603-609, EU791611-615, AY342288, and JX987796-890. GenBank accession numbers for COII sequences are: KF787572-787631 and FJ860250-251; GenBank accession numbers for d-loop sequences are:KF527299-408. GenBank accession numbers for ITS2 sequences are: JN866835-JN866905, JN866908-910, and KF527286-298. Sequence alignments (concatenated mtDNA and mtDNA + ITS2) and the corresponding ML trees are accessible through TreeBase (URL: http://purl.org/phylo/treebase/phylows/study/TB2:S14894).

## Competing interests

The authors declare that they have no competing interests.

## Authors’ contributions

LB and EJB designed the study, generated, analyzed, and interpreted data. Part of this study was the object of EJB’s master thesis project. LB, DBB, MBL, AG, SN, and LD participated in the conception of the study, acquisition and interpretation of data. AGC, CGC, RL, and JLHF were instrumental in data acquisition. All authors read and approved the final manuscript.

## Supplementary Material

Additional file 1 **12SrDNA haplotype distribution.** Haplotype distribution for 12SrDNA sequences. TX = Texas (U.S.), MX = Mexico, CR = Costa Rica, EC = Ecuador, CO = Colombia, FG = French Guiana, RO = Rondonia (Brazil, AF-MG = Atlantic Forest and Mato Grosso (Brazil), Yu = Yungas (Argentina), PA = Paraguay, CHS- CHO = Chaco Serrano and Chaco Occidental (Argentina), PE = Perú. Gray areas delimit haplotypes found clustered in the same TCS networks and phylogenetic clades. Click here for file

Additional file 2 **COII haplotype distribution.** Haplotype distribution for COII sequences. TX = Texas (U.S.), MX = Mexico, CR = Costa Rica, EC = Ecuador, CO = Colombia, FG = French Guiana, RO = Rondonia (Brazil, AF-MG = Atlantic Forest and Mato Grosso (Brazil), Yu = Yungas (Argentina), PA = Paraguay, CHS- CHO = Chaco Serrano and Chaco Occidental (Argentina), PE = Perú. Gray areas delimit haplotypes found clustered in the same TCS networks and phylogenetic clades. Click here for file

Additional file 3 **DL haplotype distribution.** Haplotype distribution for DL sequences. TX = Texas (U.S.), MX = Mexico, CR = Costa Rica, EC = Ecuador, CO = Colombia, FG = French Guiana, RO = Rondonia (Brazil, AF-MG = Atlantic Forest and Mato Grosso (Brazil), Yu = Yungas (Argentina), PA = Paraguay, CHS- CHO = Chaco Serrano and Chaco Occidental (Argentina), PE = Perú. Gray areas delimit haplotypes found clustered in the same TCS networks and phylogenetic clades. Click here for file

Additional file 4 **ITS2 genotype distribution.** Genotype distribution for ITS2 sequences. LTX = Texas (U.S.), MX = Mexico, CR = Costa Rica, EC = Ecuador, CO = Colombia, FG = French Guiana, RO = Rondonia (Brazil, AF-MG = Atlantic Forest and Mato Grosso (Brazil), Yu = Yungas (Argentina), PA = Paraguay, CHS- CHO = Chaco Serrano and Chaco Occidental (Argentina), PE = Perú. Gray areas delimit haplotypes found clustered in the same TCS networks and phylogenetic clades. Click here for file

Additional file 5 **12SrDNA maximum likelihood tree.** Tree representing the relationships between *A. cajennense* inferred by ML analysis of 12SrDNA gene sequences. NW = Texas, Mexico, Cost Rica, Ecuador clade, NE = French Guiana and Rondonia (Brazil) clade, CO = Colombia, EA = Yungas Argentina + Atlantic Forest of Brazil, AR = Chaco (Argentina and Paraguay), PE = inter-Andean Valley of Perú. Numbers over the branches represent MP bootstrap values (1000 replicates), ML bootstrap values (100 replicates), and BA posterior probabilities respectively. (B) Unrooted TCS Network (95% parsimony cut-off). Same colors in A and B represent the same samples. Click here for file

Additional file 6 **COII maximum likelihood tree.** Tree representing the relationships between A. cajennense inferred by ML analysis of COII gene sequences. NW = Texas, Mexico, Cost Rica, Ecuador clade, NE = French Guiana and Rondonia (Brazil) clade, CO = Colombia, EA = Yungas Argentina + Atlantic Forest of Brazil, AR = Chaco (Argentina and Paraguay), PE = inter-Andean Valley of Perú. Numbers over the branches represent MP bootstrap values (1000 replicates), ML bootstrap values (100 replicates), and BA posterior probabilities respectively. (B) Unrooted TCS Network (95% parsimony cut-off). Same colors in A and B represent the same samples. Click here for file

Additional file 7 **DL maximum likelihood tree.** Tree representing the relationships between A. cajennense inferred by ML analysis of DL sequences. NW = Texas, Mexico, Cost Rica, Ecuador clade, NE = French Guiana and Rondonia (Brazil) clade, CO = Colombia, EA = Yungas Argentina + Atlantic Forest of Brazil, AR = Chaco (Argentina and Paraguay), PE = inter-Andean Valley of Perú. Numbers over the branches represent MP bootstrap values (1000 replicates), ML bootstrap values (100 replicates), and BA posterior probabilities respectively. (B) Unrooted TCS Network (95% parsimony cut-off). Same colors in A and B represent the same samples. Click here for file

Additional file 8 **ITS2 maximum likelihood tree.** Tree representing the relationships between A. cajennense inferred by ML analysis of ITS2 sequences. NW = Texas, Mexico, Cost Rica, Ecuador clade, NE = French Guiana and Rondonia (Brazil) clade, CO = Colombia, EA = Yungas Argentina + Atlantic Forest of Brazil, AR = Chaco (Argentina and Paraguay), PE = inter-Andean Valley of Perú. Numbers over the branches represent MP bootstrap values (1000 replicates), ML bootstrap values (100 replicates), and BA posterior probabilities respectively. (B) Unrooted TCS Network (95% parsimony cut-off). Same colors in A and B represent the same samples. Click here for file
