# Supplementary material for: Amblyomma cajennense (Fabricius, 1787) (Acari: Ixodidae), the Cayenne tick: phylogeography and evidence for allopatric speciation
Source: BMC Evol Biol. 2013 Dec 9;13:267. doi: 10.1186/1471-2148-13-267 (PMC3890524; doi:10.1186/1471-2148-13-267)
Supplement: Additional file 5 — 12SrDNA maximum likelihood tree. Tree representing the relationships between A. cajennense inferred by ML analysis of 12SrDNA gene sequences. NW = Texas, Mexico, Cost Rica, Ecuador clade, NE = French Guiana and Rondonia (Brazil) clade, CO = Colombia, EA = Yungas Argentina + Atlantic Forest of Brazil, AR = Chaco (Argentina and Paraguay), PE = inter-Andean Valley of Perú. Numbers over the branches represent MP bootstrap values (1000 replicates), ML bootstrap values (100 replicates), and BA posterior probabilities respectively. (B) Unrooted TCS Network (95% parsimony cut-off). Same colors in A and B represent the same samples. [file 1471-2148-13-267-S5.pptx]

## Slide 1
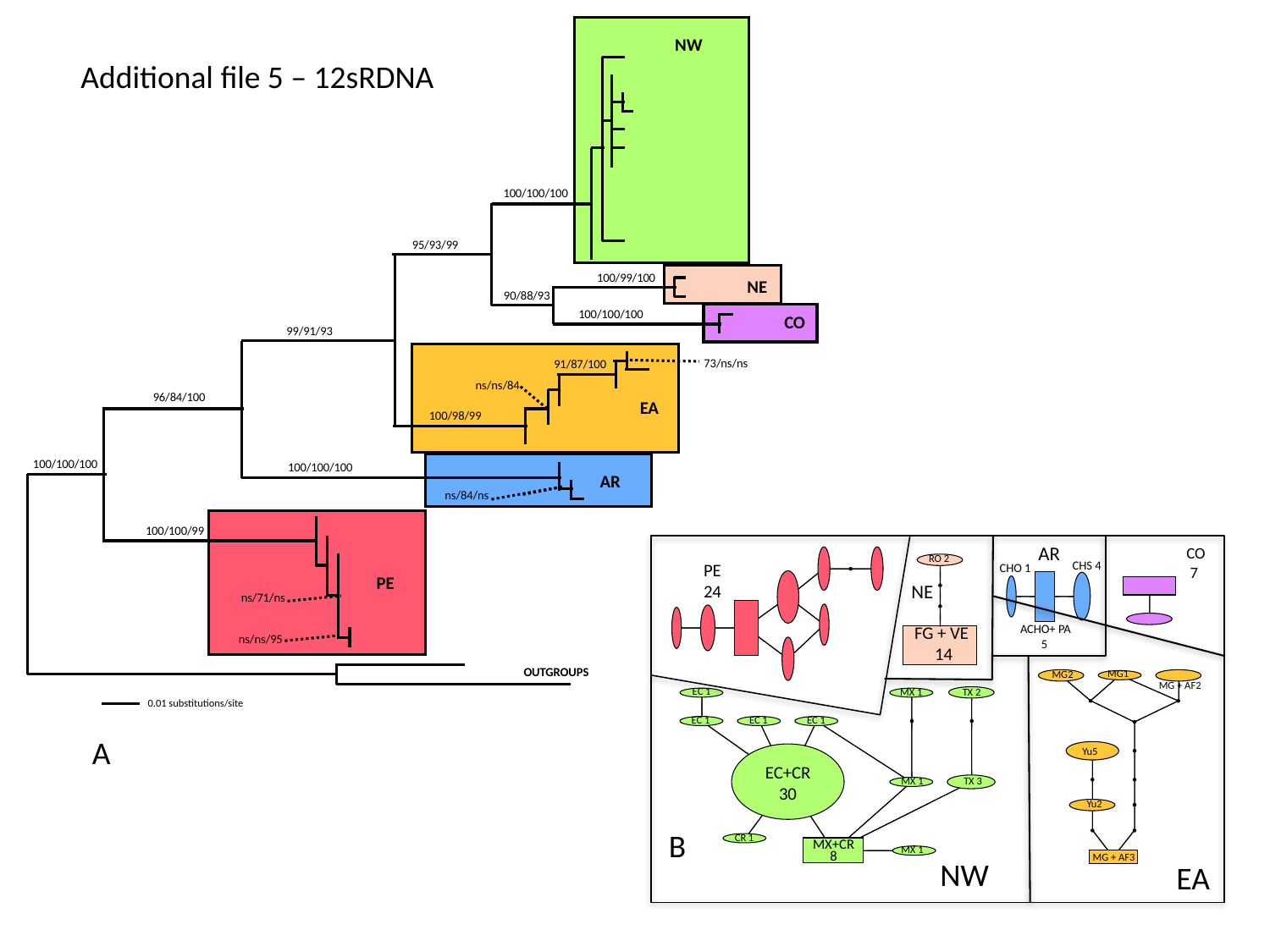

NW
Additional file 5 – 12sRDNA
100/100/100
95/93/99
100/99/100
NE
90/88/93
100/100/100
CO
99/91/93
73/ns/ns
91/87/100
ns/ns/84
96/84/100
EA
100/98/99
100/100/100
100/100/100
AR
ns/84/ns
100/100/99
AR
EC+CR
30
CO
 7
RO 2
CHS 4
PE
24
CHO 1
PE
NE
ns/71/ns
ACHO+ PA
5
FG + VE
 14
ns/ns/95
OUTGROUPS
MG1
MG2
MG + AF2
EC 1
MX 1
TX 2
0.01 substitutions/site
EC 1
EC 1
EC 1
A
Yu5
TX 3
MX 1
Yu2
B
CR 1
MX+CR
8
MX 1
MG + AF3
NW
EA
