# Supplementary figures and images for: Amblyomma cajennense (Fabricius, 1787) (Acari: Ixodidae), the Cayenne tick: phylogeography and evidence for allopatric speciation
Source: BMC Evol Biol. 2013 Dec 9;13:267. doi: 10.1186/1471-2148-13-267 (PMC3890524; doi:10.1186/1471-2148-13-267)

## Slide 1
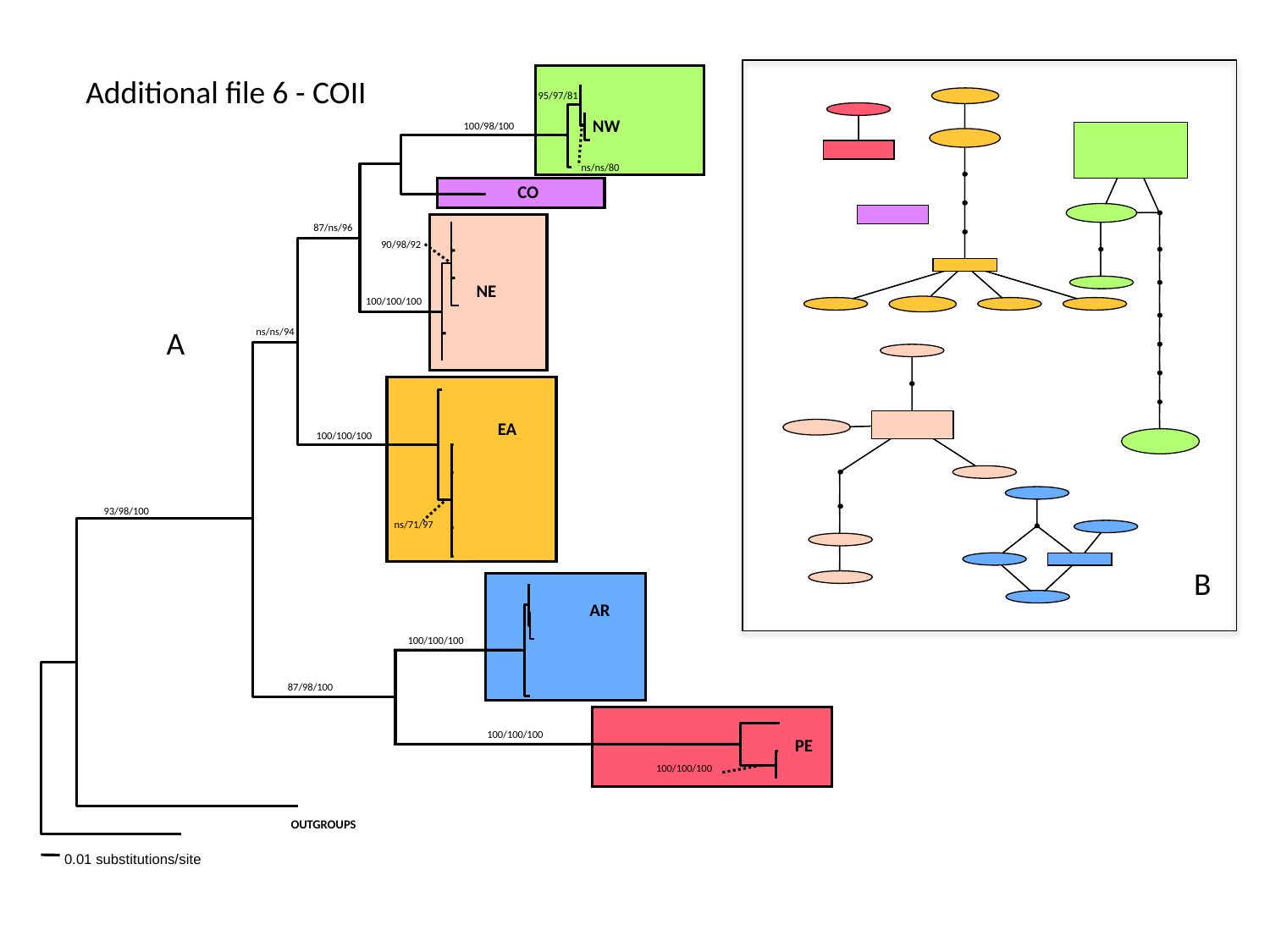

Additional file 6 - COII
95/97/81
NW
100/98/100
ns/ns/80
CO
87/ns/96
90/98/92
NE
100/100/100
A
ns/ns/94
EA
100/100/100
93/98/100
ns/71/97
B
AR
100/100/100
87/98/100
100/100/100
PE
100/100/100
OUTGROUPS
0.01 substitutions/site

Supplement: Additional file 6 — COII maximum likelihood tree. Tree representing the relationships between A. cajennense inferred by ML analysis of COII gene sequences. NW = Texas, Mexico, Cost Rica, Ecuador clade, NE = French Guiana and Rondonia (Brazil) clade, CO = Colombia, EA = Yungas Argentina + Atlantic Forest of Brazil, AR = Chaco (Argentina and Paraguay), PE = inter-Andean Valley of Perú. Numbers over the branches represent MP bootstrap values (1000 replicates), ML bootstrap values (100 replicates), and BA posterior probabilities respectively. (B) Unrooted TCS Network (95% parsimony cut-off). Same colors in A and B represent the same samples. [file 1471-2148-13-267-S6.pptx]

## Slide 1
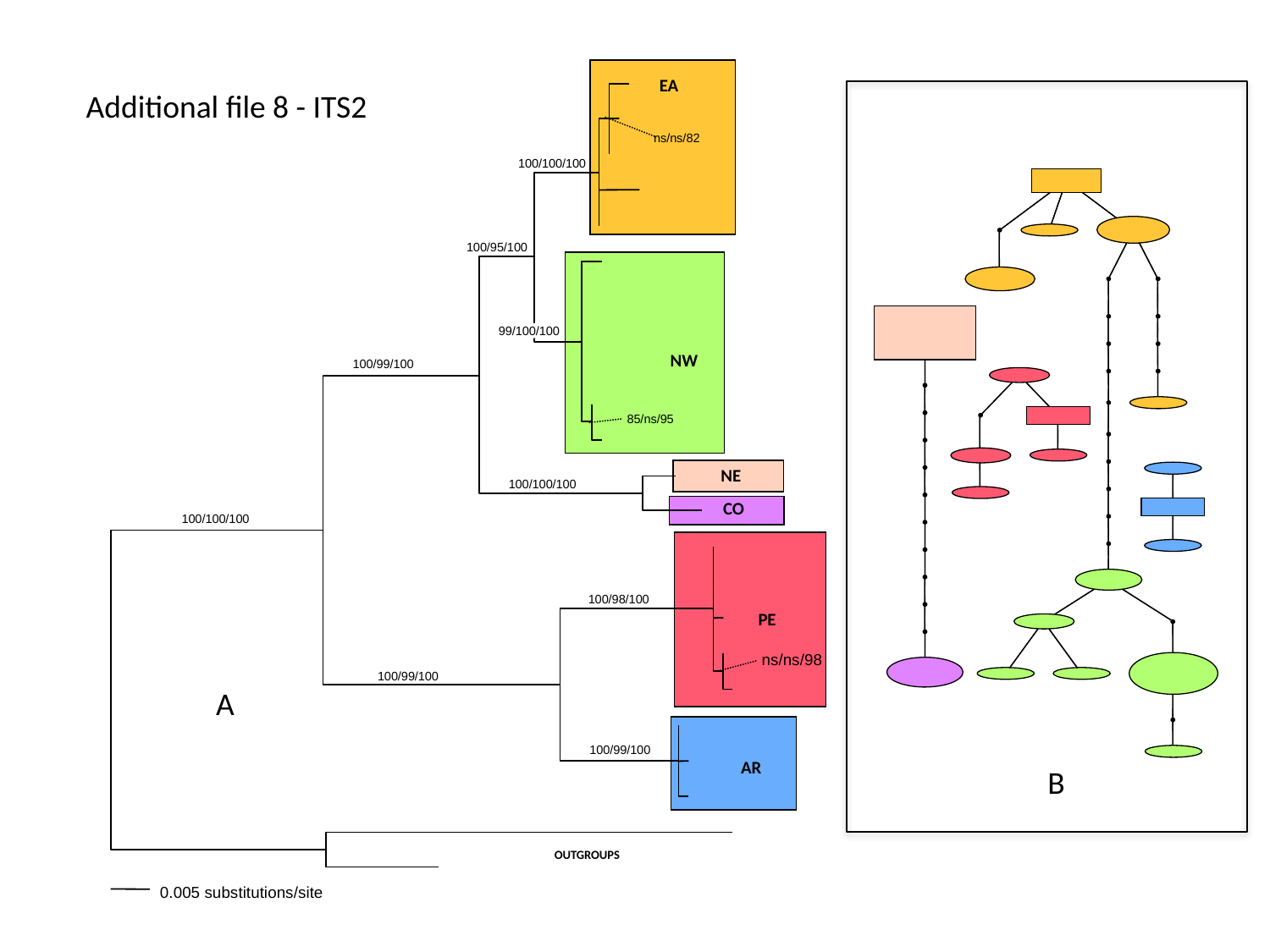

EA
Additional file 8 - ITS2
ns/ns/82
100/100/100
100/95/100
99/100/100
NW
100/99/100
85/ns/95
NE
100/100/100
CO
100/100/100
100/98/100
PE
ns/ns/98
100/99/100
A
100/99/100
AR
B
OUTGROUPS
0.005 substitutions/site

Supplement: Additional file 8 — ITS2 maximum likelihood tree. Tree representing the relationships between A. cajennense inferred by ML analysis of ITS2 sequences. NW = Texas, Mexico, Cost Rica, Ecuador clade, NE = French Guiana and Rondonia (Brazil) clade, CO = Colombia, EA = Yungas Argentina + Atlantic Forest of Brazil, AR = Chaco (Argentina and Paraguay), PE = inter-Andean Valley of Perú. Numbers over the branches represent MP bootstrap values (1000 replicates), ML bootstrap values (100 replicates), and BA posterior probabilities respectively. (B) Unrooted TCS Network (95% parsimony cut-off). Same colors in A and B represent the same samples. [file 1471-2148-13-267-S8.pptx]
