# Supplementary material for: Amblyomma cajennense (Fabricius, 1787) (Acari: Ixodidae), the Cayenne tick: phylogeography and evidence for allopatric speciation
Source: BMC Evol Biol. 2013 Dec 9;13:267. doi: 10.1186/1471-2148-13-267 (PMC3890524; doi:10.1186/1471-2148-13-267)
Supplement: Additional file 7 — DL maximum likelihood tree. Tree representing the relationships between A. cajennense inferred by ML analysis of DL sequences. NW = Texas, Mexico, Cost Rica, Ecuador clade, NE = French Guiana and Rondonia (Brazil) clade, CO = Colombia, EA = Yungas Argentina + Atlantic Forest of Brazil, AR = Chaco (Argentina and Paraguay), PE = inter-Andean Valley of Perú. Numbers over the branches represent MP bootstrap values (1000 replicates), ML bootstrap values (100 replicates), and BA posterior probabilities respectively. (B) Unrooted TCS Network (95% parsimony cut-off). Same colors in A and B represent the same samples. [file 1471-2148-13-267-S7.pptx]

## Slide 1
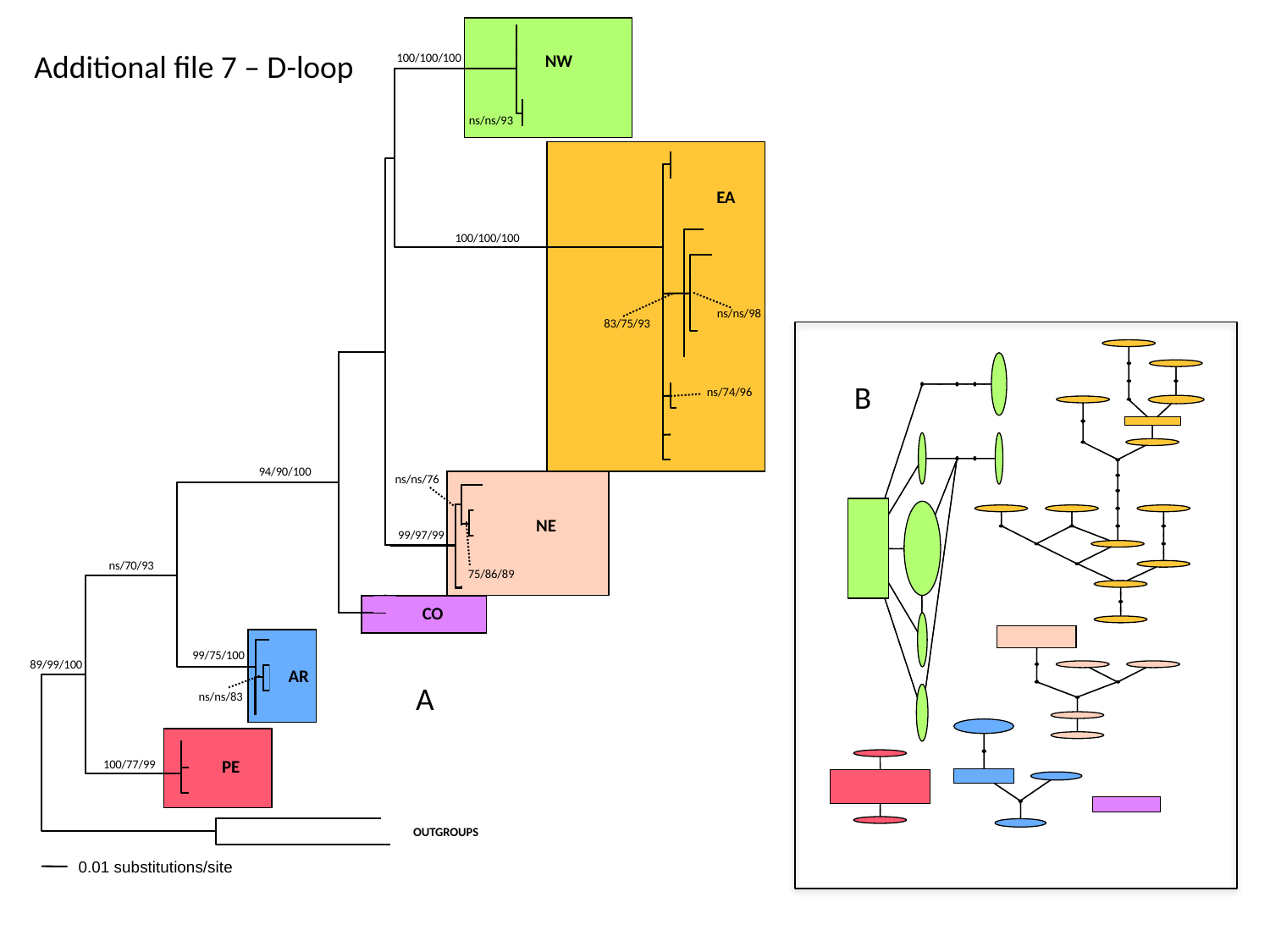

Additional file 7 – D-loop
NW
100/100/100
ns/ns/93
EA
100/100/100
ns/ns/98
83/75/93
B
ns/74/96
94/90/100
ns/ns/76
NE
99/97/99
ns/70/93
75/86/89
CO
99/75/100
89/99/100
AR
A
ns/ns/83
PE
100/77/99
OUTGROUPS
0.01 substitutions/site
